# Prevalence of chronic pain syndrome in patients who have undergone hallux valgus percutaneous surgery: a comparison of sciatic-femoral and ankle regional ultrasound-guided nerve blocks

**DOI:** 10.1186/s12891-021-04911-4

**Published:** 2021-12-15

**Authors:** Carlo Biz, Gianfranco de Iudicibus, Elisa Belluzzi, Miki Dalmau-Pastor, Nicola Luigi Bragazzi, Manuela Funes, Gian-Mario Parise, Pietro Ruggieri

**Affiliations:** 1 Orthopedics and Orthopedic Oncology, Department of Surgery, Oncology and Gastroenterology DiSCOG, University of Padova, via Giustiniani 3, 35128 Padova, Italy; 2 Minimally Invasive Foot and Ankle Society (MIFAS By Grecmip), 2 Rue Georges Negrevergne, 33700 Merignac, France; 3 Musculoskeletal Pathology and Oncology Laboratory, Orthopaedics and Orthopedics Oncology, Department of Surgery, Oncology and Gastroenterology DiSCOG, University of Padova, via Giustiniani 3, 3518 Padova, Italy; 4 Human Anatomy and Embryology Unit, Department of Pathology and Experimental Therapeutics, School of Medicine and Health Sciences, University of Barcelona, Barcelona, Spain; 5 Laboratory for Industrial and Applied Mathematics, Department of Mathematics and Statistics, York University, Toronto, Canada; 6 Institute of Anesthesia and Reanimation, Department of Medicine DIMED, University of Padova, Padova, Italy

**Keywords:** Chronic pain, Postoperative pain, Hallux valgus, Foot surgery, Minimally invasive surgery, Anaesthesia, Ankle block, Femoral-sciatic block

## Abstract

**Background:**

Chronic pain syndrome (CPS) is a common complication after operative procedures, and only a few studies have focused on the evaluation of CPS in foot-forefoot surgery and specifically on HV percutaneous correction. The objective of this study was to compare postoperative pain levels and incidence of CPS in two groups of patients having undergone femoral-sciatic nerve block or ankle block regional anaesthesia before hallux valgus (HV) percutaneous surgery and the association between postoperative pain levels and risk factors between these patient groups.

**Methods:**

A consecutive patient series was enrolled and evaluated prospectively at 7 days, 1, 3 and 6 months after surgery. The participants were divided into two groups according to the regional anaesthesia received, femoral-sciatic nerve block or ankle block, and their outcomes were compared. The parameters assessed were postoperative pain at rest and during movement by the numerical rating scale (NRS), patient satisfaction using the Visual Analogue Scale (VAS), quality of life and return to daily activities. Statistical analysis was performed.

**Results:**

One hundred fifty-five patients were assessed, 127 females and 28 males. Pain at rest (*p* < 0.0001) and during movement (*p* < 0.0001) significantly decreased during the follow-ups; at 6 months, 13 patients suffered from CPS. Over time, satisfaction remained stable (*p* > 0.05), quality of life significantly increased and patients returned to daily activities and work (*p* < 0.0001). No significant impact of type of anaesthesia could be detected. ASA 3 (*p* = 0.043) was associated to higher pain during movement; BMI (*p* = 0.005) and lumbago (*p* = 0.004) to lower satisfaction. No operative-anaesthetic complications were recorded. Postoperative pain at rest and during movement improved over time independently of the regional block used, with low incidence of CPS at last follow-up. Among risk factors, only a higher ASA was associated to higher pain during movement, while higher BMI and lumbago to lower satisfaction.

**Conclusions:**

Both ultrasound-guided sciatic-femoral and ankle blocks were safe and effective in reducing postoperative pain with low incidence of CPS at last follow-up.

**Trial registration:**

Clinical Trial NCT02886221. Registered 1 September 2016.

## Background

Chronic pain syndrome (CPS) is a common complication after operative procedures, which can lead to a significant disease burden and reduced quality of life in affected individuals [1]. Overall, the estimated incidence of persistent disabling pain after surgery is in the range of 10–50% [2]. The first paper on CPS was published by Crombie et al. [3] in 1998, and the first accepted definition was proposed by Macrae in 2001 [4]: *“CPS is a persistent pain that has developed after a surgical procedure, of at least 2 months duration and for which other causes (malignancy, chronic infection or a continuation of a pre-existing problem) have been excluded.”*

This definition was later revised and implemented by the International Association of Pain Study (IAPS) [5]. Currently, 3 months are accepted as the minimal duration for the diagnosis of CPS [5], while its minimum intensity should be ≥4 on a 0–10 numeric rating scale (NRS) [6].

Different anaesthesiological techniques adopted also seem to have a potential influence on postoperative pain and CPS genesis [7]. In orthopaedic surgery, general and spinal anaesthesia are often used, but they can cause postoperative complications such as nausea, vomiting, urinary retention, bowel motility alteration, back pain and/or headache [8–12]. Currently, with the increasing use of ultrasonography for guidance of peripheral nerve blocks, regional anaesthesia has become the most popular method for foot and ankle surgery, and in particular for elective orthopaedic forefoot operative procedures, such as hallux valgus (HV) correction [13, 14]. Several studies have shown peripheral nerve blocks to be highly effective for patients having in-patient forefoot surgery, both in delaying the onset of pain and reducing pain in the early postoperative period [14, 15].

A recent report has shown that ultrasound-guided *femoral-sciatic nerve block* is associated with satisfactory anaesthesia without pre- and postoperative complications, besides providing postoperative pain control for an average of 12 h [16]. Nevertheless, the use of peripheral nerve blocks still holds some disadvantages such as theoretically increased risk of accidental injury in the early postoperative period due to transient weakness and an insensate lower extremity [17]. *Ankle block* is an attractive alternative to *femoral-sciatic nerve block* for primary anaesthesia for forefoot procedures that may reduce potential risks associated with a more proximal nerve block [18]. While most of studies in the literature describe CPS incidence after breast surgery, thoracotomy, amputation, abdominal surgery and other surgeries [1], very few studies focus on the evaluation of postoperative pain and CPS after foot-forefoot surgery and specifically on HV percutaneous correction [19, 20].

Hence, the primary aim of this prospective study was to evaluate postoperative pain levels and incidence of CPS in patients who underwent ultrasound-guided *femoral-sciatic nerve block* or *ankle block* before HV percutaneous operative procedure performed as outpatient surgery. The secondary aim was to assess the association between postoperative pain levels and the risk factors between these two groups of patients.

## Materials and methods

### Patients

At our institution, between May 2018 and July 2020, a consecutive series of adult, Caucasian patients with diagnosis of symptomatic Hallux Valgus (HV), resistant to at least six-month conservative treatment (including stretching, mobilisation, manipulation, shoe modifications, orthoses, splints or night splinting, medial bunion pads, local ice and general analgesics [21], was enrolled in this prospective, non-randomised, single-centre and single surgeon cohort study. The study protocol was approved by the Local Ethics Committee of Padova (4065/AO/17), registered with ClinicalTrials.gov (NCT02886221 01/09/2016) and conducted according to good clinical practice guidelines and the ethical standards of the 1964 Declaration of Helsinki as revised in 2000. The subjects participating in this study received a thorough explanation of the risks and benefits of inclusion and gave their oral and written informed consent to publish the data.

According to the indications of our institutional forefoot operative protocol, a percutaneous surgery such as Reverdin-Isham and Akin osteotomies associated with lateral soft-tissue release was performed for the correction of mild-to-moderate HV deformity [22]. The classification of the HV deformity was based on the presence of one of the following Mann and Coughlin parameters [23]: mild HV was defined as an intermetatarsal angle (IMA) ≤ 11° and a metatarsophalangeal hallux valgus angle (HVA) < 20°, and less than 50% subluxation of the medial sesamoid (grade 1); moderate HV was defined as an IMA > 11 degrees but < 16 degrees and a HVA of 20 ° to 40 °, with 50 to 75% subluxation of tibial sesamoid (grade 2).

All forefoot procedures were performed in the morning (8:00–2:00) in outpatient surgery by the same experienced surgeon, the senior author, trained in minimally invasive surgery (MIS).

Inclusion criteria for the study population were patients undergoing outpatient, elective, unilateral, only percutaneous surgery as previously indicated [22] and only on their first ray for mild-to-moderate HV (without concomitant forefoot procedures: e.g. hammertoe correction, claw toe correction).

Exclusion criteria were as follows: use of peripheral blocks different from ankle-block or sciatic-femoral block, continuous nerve blocks, history of allergy to local anaesthetic, previous dry needling or local corticosteroid injections, bilateral HV, arthritis and stiffness of metatarsophalangeal joint, previous trauma, foot and ankle surgery, congenital deformities of the foot, hallux valgus and rigidus, hypermobility of first ray, Freiberg infraction, metatarsalgia and Morton’s neuroma, and diagnosis of rheumatic, metabolic (diabetes), neurologic (prior nerve injury, sciatica, peripheral neuropathy), infective or psychiatric pathologies (bipolar disorder, schizophrenia, dementia and developmental disorders including autism). These strict selection criteria were used to avoid possible confounding factors, which could have impacted the generalisability of our results. Specifically, we excluded those conditions responsible for chronic pain or altered perception of pain in the foot.

### Regional anaesthesia procedures

Two different types of ultrasound-guided regional anaesthesia were performed: sciatic-femoral block and ankle-block. All regional block procedures were performed by one of the three senior anaesthetists of the same experienced anaesthesiological team of our Orthopaedic Department. Both nerve blocks were performed with ultrasound guidance with or without the use of a neurostimulator for sciatic-femoral block and ankle-block, respectively, and employed after positioning the patient in a supine decubitus position. During the two-year study period, both regional block procedures were chosen without any technique preference by the same anaesthesiological team and alternated every week according to the study protocol. Hence, patients were allocated into 2 groups according to the type of block used: sciatic-femoral block and ankle block.

To improve patient cooperation and comfort, standard premedication was administered using intravenous Midazolam (1–2 mg) and Fentanyl (0.1 mg). Intra-operative sedation was obtained using Propofol (Diprivan) 1.5 mg/kg to 2.5 mg/kg for induction and a continuous infusion of 4–8 mg/kg/h for maintenance. Finally, no intraoperative morphine and/or nonsteroidal anti-inflammatory (NSAID) was given during the operative procedure, according to routine practice.

#### Sciatic-femoral nerve block technique

**Fig. 1:**
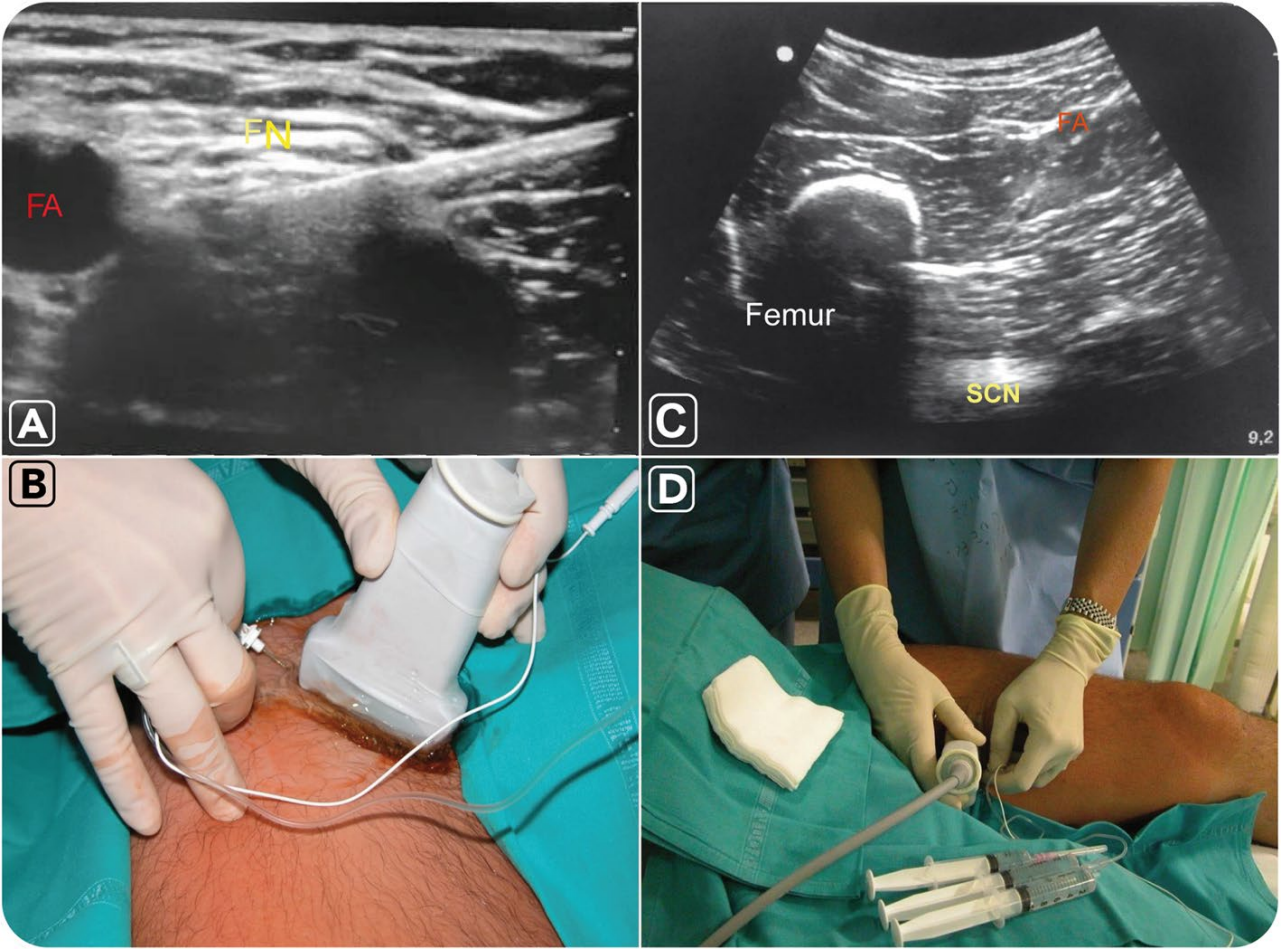
Ultrasound (**A**-**C**) and clinical (**B**-**D**) images of *femoral-sciatic nerve block procedures* with the patient lying in a supine position. For *femoral block* (**A**-**B**), using an ultrasound-guided technique (**A**), the needle is advanced through the fascia lata and iliaca until an adequate position with respect to the femoral nerve (FN) is reached. The site of needle insertion (**B**) is located at the femoral crease but below the inguinal crease and immediately lateral to the pulse of the femoral artery (FA). For *sciatic block* (**C**-**D**), using an ultrasound-guided technique (**C**), the sciatic nerve (SCN) is seen as a hyperechoic oval structure sandwiched between the adductor magnus muscle and the hamstring muscles. The nerve is typically visualised at a depth of 6–8 cm, under the femoral artery (FA), the femur and the adductor magnus muscle. The needle is inserted in plane from the medial aspect of the thigh and advanced toward the sciatic nerve (**D**)

Sciatic-femoral nerve block was performed via anterior approach, (Fig. 1).

For femoral block (A-B), using an ultrasound-guided technique (A), the needle is advanced through the fascia lata and iliaca until an adequate position with respect to the femoral nerve (FN) is reached. The site of needle insertion (B) is located at the femoral crease, below the inguinal crease and immediately lateral to the pulse of the femoral artery (FA).

For sciatic block (C-D), the sciatic nerve (SCN) is seen as a hyperechoic oval structure sandwiched between the adductor magnus muscle and the hamstring muscles, typically visualised at a depth of 6–8 cm, under the femoral artery (FA), the femur and the adductor magnus muscle. The femoral block is performed by inserting a 22-gauge needle connected to a nerve stimulator set at a current intensity of 1 mA (0.1 ms/2 Hz), 1.5–2 cm lateral to the femoral artery and 1–2 cm distal to an inguinal ligament in a cephalic direction at a 30–45° angle. As the quadriceps muscle contractions are obtained, the current is gradually decreased while the needle is advanced. The position of the needle is adequate when patellar twitches are elicited with current output between 0.3 and 0.5 mA. The drug is then injected (Fig. 1A and B). For the sciatic block, a 21-gauge needle is introduced at a perpendicular angle to the skin plane. When nerve stimulation is used (0.5 mA, 0.1 ms), the contact of the needle tip with the nerve usually is associated with a motor response of the calf or foot. Then, 20 mL of Ropivacaine 0.75% is injected (Fig. 1C and D).

#### Ankle block technique

**Fig. 2:**
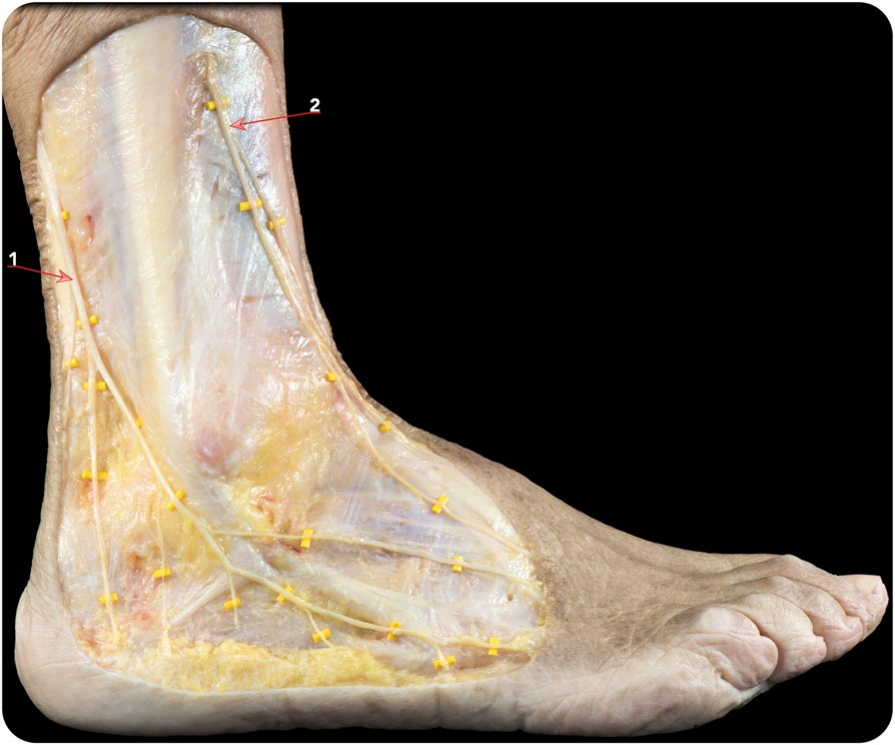
Anatomical dissection image of the anterolateral aspect of the lower leg and ankle demonstrating the anatomy of the nerves of the lateral compartment of the ankle involved in the ankle blocks: (1) the sural nerve and (2) the superficial peroneal nerve. The dissection shows the distal division of the sural nerve into several branches along the lateral aspect of the ankle and foot and the two branches of the superficial peroneal nerve (the medial and intermediate dorsal cutaneous nerves)

**Fig. 3:**
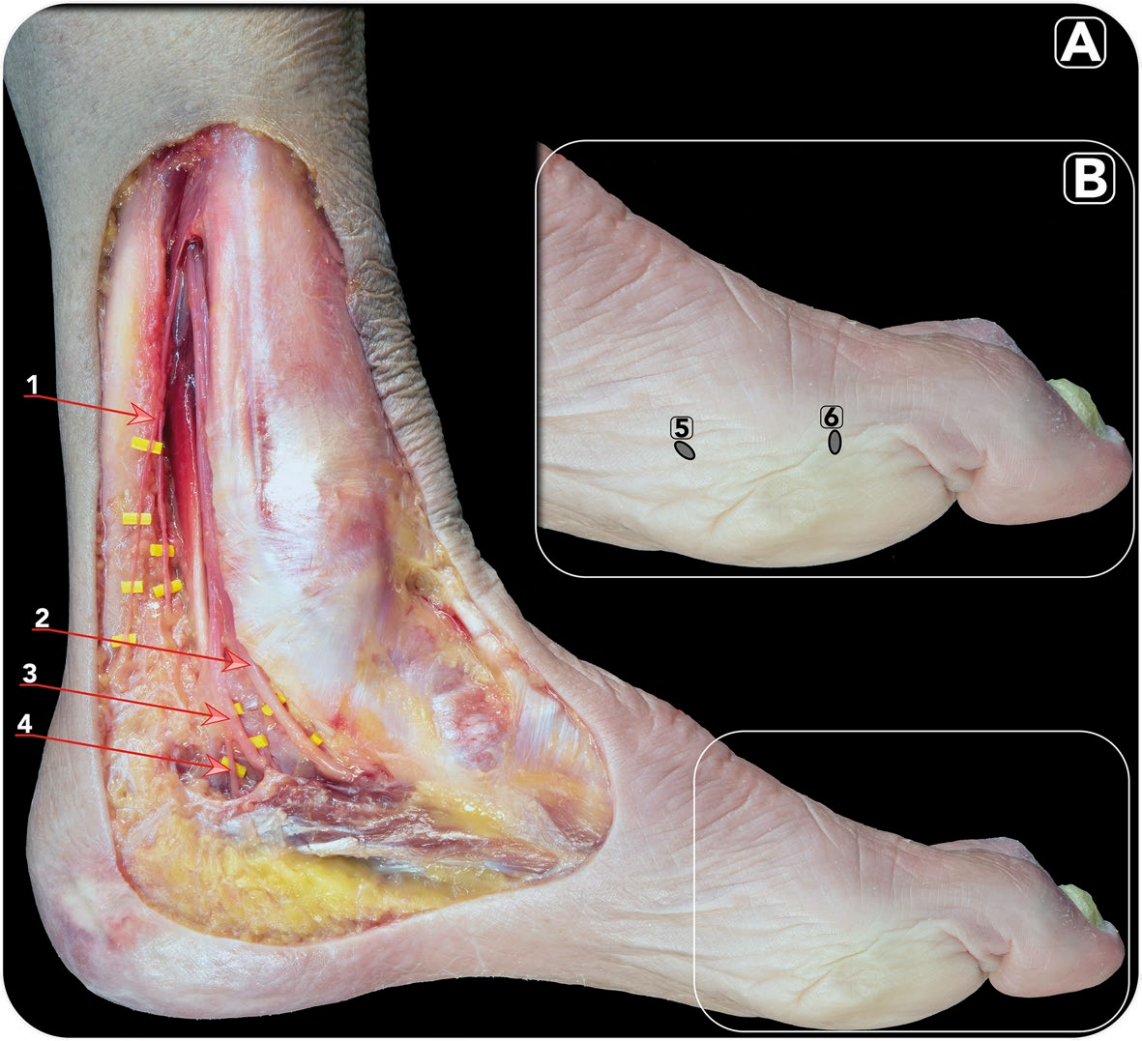
Anatomical dissection image (**A**) of the region of the tarsal tunnel demonstrating the anatomy of the nerves of the medial compartment of the ankle involved in the ankle blocks: the tibial nerve: (1) Medial calcaneal branches; (2) Medial plantar nerve; (3) Lateral plantar nerve; (4) Inferior calcaneal nerve (Baxter’s nerve). On the right, detail (**B**) of the first metatarsophalangeal joint showing the percutaneous entry points for (5) the first metatarsal distal osteotomy (Reverdin-Isham) and for (6) the first phalanx osteotomy (Akin)

**Fig. 4:**
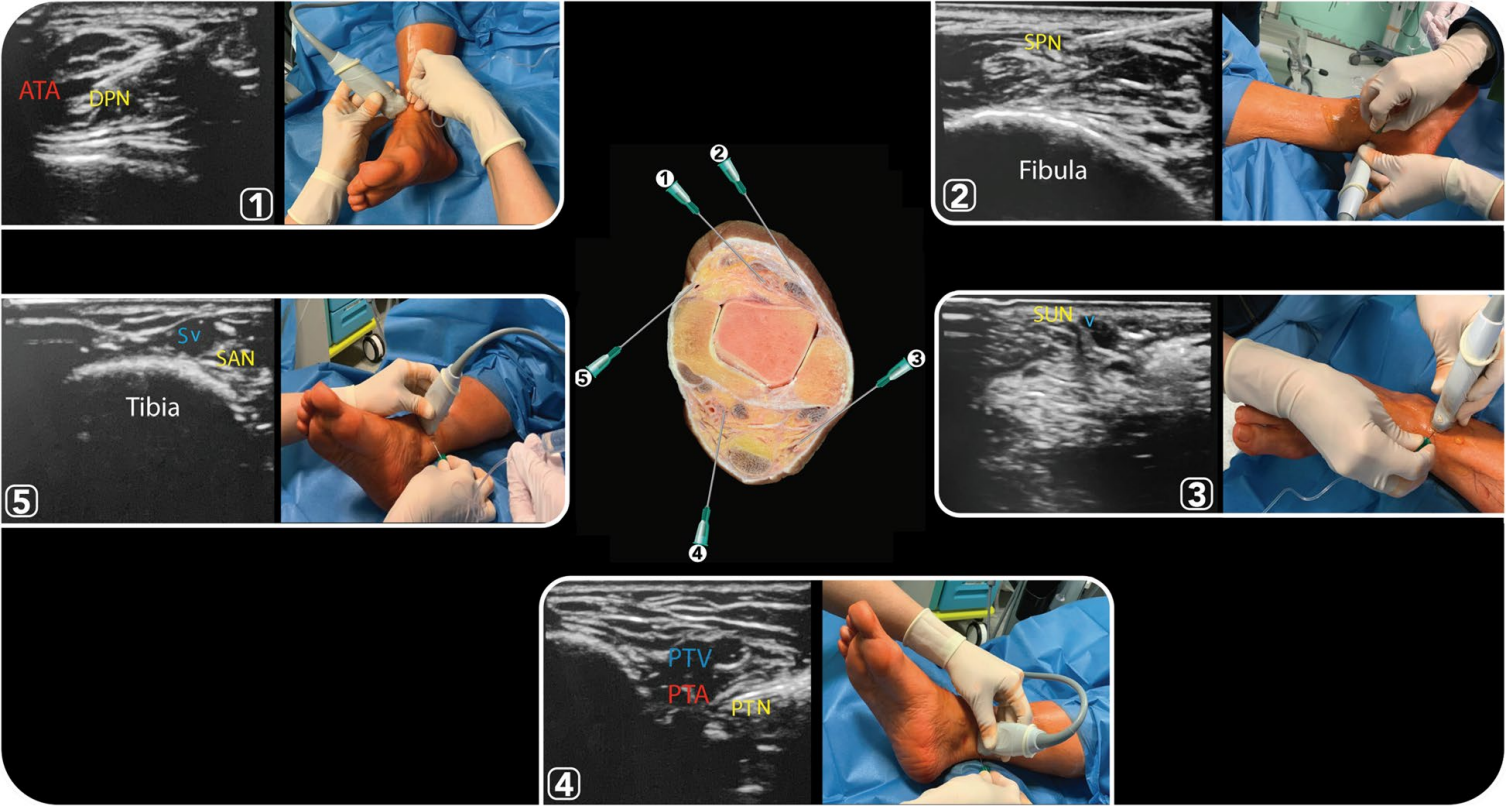
Anatomical, ultrasound and clinical images of ankle block procedures, which involve anaesthetising five separate nerves: two deep (posterior tibial and deep peroneal) and three superficial nerves (superficial peroneal, sural and saphenous). (1) Deep Peroneal Nerve: it innervates the ankle extensor muscles, the ankle joint and the web space between the first and second toes. A transducer placed in the transverse orientation at the level of the extensor retinaculum will show this nerve (DPN) lying immediately lateral to the anterior tibial artery (ATA) on the surface of the tibia. (2) Superficial Peroneal Nerve: it innervates the dorsum of the foot and emerges to lie superficial to the fascia, 10–20 cm above the ankle joint on the anterolateral surface of the leg, and divides into two or three small branches. A transducer placed transversely on the leg, approximately 5–10 cm proximal and anterior to the lateral malleolus, will identify the hyperechoic nerve branches (SPN) lying in the subcutaneous tissue immediately superficial to the fascia. (3) Sural Nerve: it innervates the lateral margin of the foot and ankle. This nerve (SUN) can be traced back along the posterior aspect of the leg, running in the midline superficial to the Achilles tendon and gastrocnemius muscles, in the immediate vicinity of the small saphenous vein (V). (4) Posterior tibial nerve: it provides innervation to the heel and sole of the foot. This nerve (N) can be seen posterior to the posterior tibial artery (PTA) and vein (PTV) using a linear transducer placed transversely at the level of the medial malleolus. The nerve typically appears hyperechoic with a honeycomb pattern. (5) Saphenous nerve: it innervates the medial malleolus and a variable portion of the medial aspect of the leg below the knee. This nerve (SAN) travels down the medial leg alongside the great saphenous vein (SV). Because it is a small nerve, it is best visualised 10–15 cm proximal to the medial malleolus using the great saphenous vein as a landmark

The ankle block involves anaesthetising the nerve supply to the foot, which consists of five separate nerves (Figs. 2 and 3A): two deep (the posterior branch of the tibial nerve and the deep peroneal nerve) and three superficial (saphenous, superficial peroneal and sural nerves). All five nerves are identified using anatomical landmarks as described by Schurman and Dhukaram and Kumar (Fig. 4) [24, 25]. This block is performed by injecting 19 ml di Ropivacaine 0.75% in amounts of 5 mL around the two deeper nerves supplying the foot and 3 ml for the superficial ones.

### Operative procedures

**Fig. 5:**
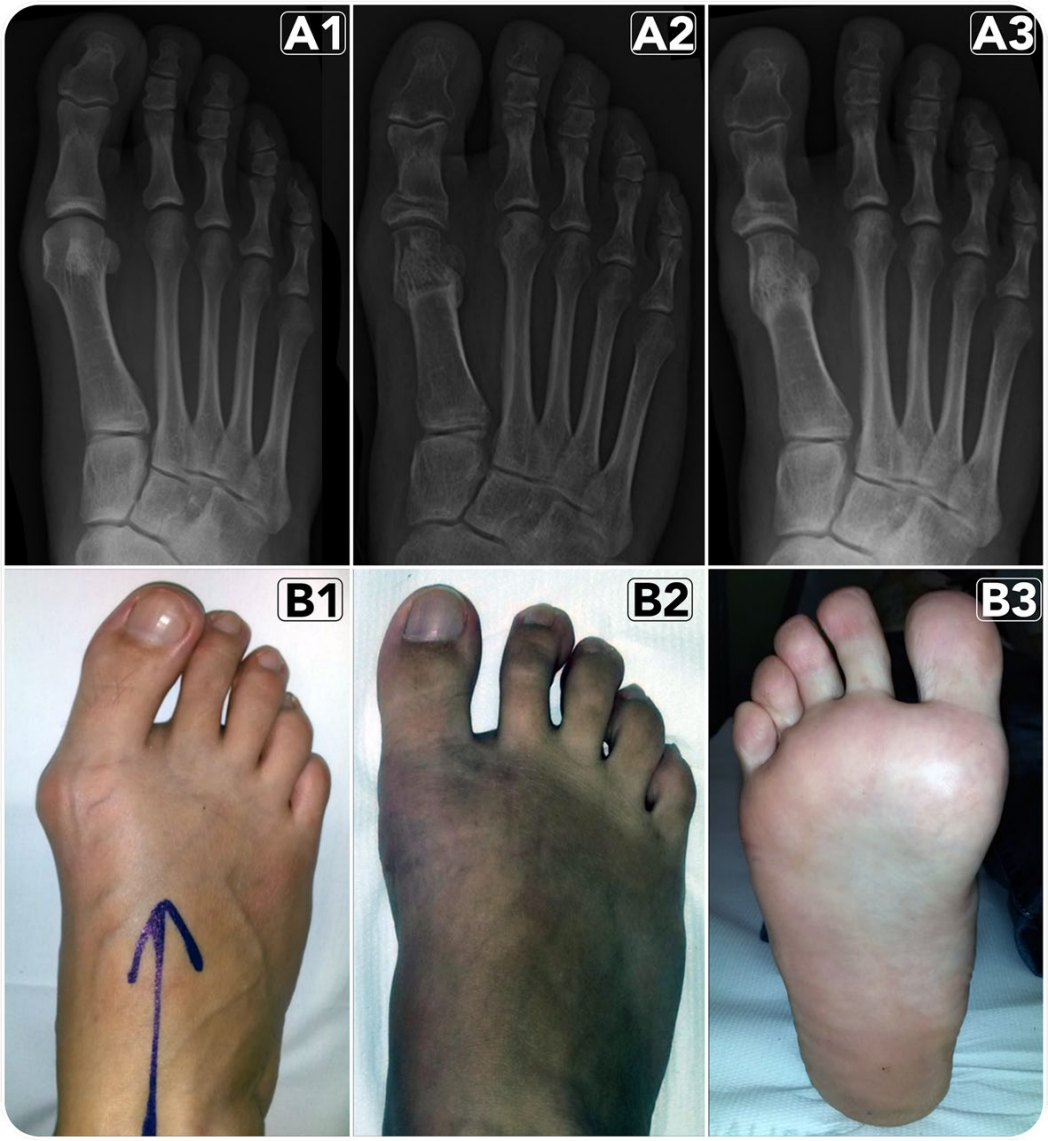
A 39-year-old woman with right mild HV after having undergone percutaneous Reverdin-Isham osteotomy, lateral release and Akin osteotomy for HV correction: (**A**) antero-posterior radiographic images at preoperative period (1), 3-month follow-up (2) and 6-month follow-up (e). (**B**) Clinical images at preoperative period (1) and at 6-month follow-up (1–2)

All patients underwent MIS by Reverdin-Isham and Akin percutaneous osteotomies for unilateral mild-to-moderate HV deformity performed without the use of ankle tourniquet hemostasis according to Prado’s technique [26] and as previously described (Fig. 5) [22]. At the plantar side of the medial border of the first metatarsal head, an incision of 3–5 mm long was made. A small scalpel was introduced within the joint capsule of the metatarso-phalangeal joint of the big toe through this medial approach. The medial capsule was separated from the exostosis by a sweeping movement, subsequently using also a rasp. The location of this incision prevents damage of the dorsomedial cutaneous nerve of the hallux. A cylindrical burr (3.1 × 15 mm) was then inserted to perform the exostosectomy: the dorsal medial prominence was removed from the first metatarsal head until a flat surface was obtained under fluoroscopic control. The bone eliminated, expressed as bone paste, was extruded manually by manual light pressure. A Shannon Isham burr (2 × 12 mm) was introduced through the same incision used for the exostosectomy and applied to the flat bone surface achieved previously at an angle of approximately 45° to the long axis of the first metatarsal bone. In this position, under fluoroscopic control, the Reverdin-Isham osteotomy was performed in dorsal-distal to plantar-proximal direction, extending until the lateral cortex, but without cutting it. The burr was slightly withdrawn at this point to preserve a few millimeters of the lateral cortex, while the osteotomy of the plantar cortex was performed completely [22]. A Wedge burr (3.1 × 13 mm or 4.1 × 13 mm, depending on the distal metaphyseal articular angle (DMAA) value) was then used to create a wedge with a medially oriented base. Osteoclasis of the preserved lateral cortex was achieved at the point of closing the wedge, modifying the orientation of the articular surface, normalising the DMAA value and adding intrinsic stability to the osteotomy by producing contact of the trabecular bone. Tenotomy of the adductor hallucis tendon and lateral capsulotomy was then performed through a small skin incision in the first web space. Finally, once lateral soft-tissue release was performed, a new incision 3 to 5 mm long on the lateral surface of the base of the proximal phalanx of the first toe was performed, just medial to the extensor tendons. The periosteum was removed from the lateral surface of the base of the proximal phalanx using a small scraper. Then, using a Wedge burr (3.1 × 13 mm), a wedge Akin osteotomy (with medial base) was performed. Also for this step, the lateral cortex was preserved. Closing of the osteotomy and osteoclasis of the lateral cortex was carried out by a forced varus movement of the toe. After completing the surgery, sutures and bandaging were applied. Patients were allowed to bear weight the day after the procedure using a rigid flat-soled orthopaedic shoe for the following 30-day period, according to the indications of our institutional forefoot postoperative protocol also used for other MI techniques [22, 27, 28].

.

### Institutional postoperative therapeutic protocol

Paracetamol (dose 1000 mg) was routinely administered intravenously 2 times after surgery before discharge from the hospital starting 2 h after the end of the procedure. No intramuscular injection of morphine sulphate or local anaesthesia was administered in the operating room neither suggested during the postoperative period.

According to the indications of our institutional forefoot postoperative protocol [22, 29], prophylactic antibiotic was administered only before surgery, and thromboembolic prophylaxis with nadroparin calcium was prescribed the same evening for a 10-day period. Standard postoperative medication starting from the day after surgery was prescribed: analgesic therapy with Etoricoxib (90 mg, 1 cp/day) in the morning for 2 weeks (also to prevent the development of heterotopic ossifications in the following months due to the presence of bone paste residues in surrounding soft tissues), in association with an anti-edemigen therapy (Leucoselect, Lymphaselect, and Bromeline: 1 cp/day) for 30 days [30].

### Postoperative outcome assessment

Demographic and clinical data such as sex, age at time of procedure, body mass index (BMI), American Society of Anesthesiologists (ASA) scale [31, 32], which globally estimates the surgical risk (1 = Normal health; 2 = Mild systemic disease; 3 = Severe systemic disease; 4 = Severe systemic disease constantly threatening life; 5 = Moribund; 6 = Brain-dead organ donor), and risk factors predisposing CPS (obesity, anxiety, depression, pain at the operative site, lumbago and proinflammatory states such as Raynaud syndrome and inflammatory bowel disease) were taken from medical records the day of surgery. For the present study, obesity was defined according to the standardized World Health Organization (WHO) criteria, utilizing a BMI of 30 kg/m^2^ as cut-off value.

All patients were followed up using a questionnaire collected by phone the first day after surgery and during the post-operative scheduled consultation at our out-patient clinic at 7 days, 1 month, 3 and 6 months after surgery by an independent investigator not directly involved in the patients’ operative treatment and blind to the patients’ allocated group.

The questionnaire was conceived to assess the postoperative pain referred by the patient by a numerical rating scale (NRS, ranging from 0 to 10 points) both at rest and during movement (dynamic); to index the overall patient satisfaction using Visual Analogue Scale (VAS), ranging from 0 to 10 points with 0 indicating no satisfaction and 10 denoting complete satisfaction for the performed block procedure; to assess the quality of life compared to preoperative conditions by self-reported global change (better/same/worse) on the basis of VR-12 physical and VR-12 mental quality of life [33]; to examine the return or not to daily activities and work. CPS was identified as NRS at rest ≥4. Finally, any postoperative complications of anaesthesia were recorded.

### Statistical analysis

The a priori power analysis was conducted using the software G*Power 3.1.9.7 for Windows. The minimum sample size required was computed selecting the following: F tests, family option, opting for between-factors, repeated measures ANOVA. In order to capture a small effect size as defined by Cohen [34], with an alpha error probability of 0.05, a power ranging from 0.8 to 0.95, with 5 time-points and a weak correlation among repeated measures (0.20), the minimum sample size varied from 87 to 134.

Before proceeding with data handling, statistical processing and manipulation, all figures were visually inspected to capture any potential outlier. Normality of data distribution was verified carrying out the D’Agostino-Pearson omnibus test. Continuous variables were computed as mean ± standard deviation with median reported when appropriate. Categorical variables were expressed as percentages.

A univariate analysis was conducted to identify eventual differences between patients under femoral nerve block and those under ankle block. Categorical variables were compared using the chi-square test, whereas continuous parameters were compared conducting Student’s t-test or its nonparametric version, based on the normality of data distribution.

A generalised linear model for repeated measures (at different time-points, namely, 1 and 5 post-operative days, and at 1, 3 and 6 months) was used. The homogeneity of covariance matrices and the independence assumptions were checked. The sphericity assumption was verified carrying out the Mauchly’s W test. In case of sphericity violation (when the ‘F’ test was significant) and with epsilon values (ε, quantitatively measuring the extent of departure from sphericity) less than 0.75, the Greenhouse-Geisser correction was adopted to properly adjust for the degrees of freedom of the interaction effect between different time points and the sample group. Otherwise (in case of ε greater than 0.75), the Huynh-Feldt correction was carried out. Effect size was estimated by computing the partial eta squared (ηp2) and interpreted using the following rule: small if < 0.06, moderate in the range 0.06–0.14 and large if > 0.14. Post-hoc tests using the Bonferroni correction for pairwise comparisons were conducted. This generalised linear model was applied for investigating changes in pain, movement with pain, satisfaction and quality of life at different time points.

To shed light on the determinants of the insurgence of CPS, a multivariate logistic regression analysis (with the “enter” method) was conducted.

Figures with *p*-values less than 0.05 were considered statistically significant. All statistical analyses were carried out with the commercial software “Statistical Package for the Social Sciences” (SPSS version 24.0 for Windows, IBM, Armonk, NY, USA). Graphs were generated by means of the commercial software MedCalc (MedCalc Statistical Software version 18.11.3, MedCalc Software bvba, Ostend, Belgium).

## Results

The recruited population included 155 patients. A femoral nerve block was used for 82 (52.9%) patients, while 73 (47.1%) received an ankle block. Demographic and clinical data of the recruited population are reported in Table 1.

**Table 1:** Main characteristics of the recruited sample of 155 patients

Parameters	Value
Age (years)	59.01 ± 12.21; 62
BMI (kg/m^2^)	26.88 ± 4.86; 27
Sex (n, %)	
Male	28 (18.1%)
Female	127 (81.9%)
ASA classification (n, %)	
1	47 (30.3%)
2	93 (60.0%)
3	15 (9.7%)
Risk factors (n, %)	
Preoperative Pain	73 (47.1%)
Anxiety-depression	24 (15.5%)
Inflammation	7 (4.5%)
Obesity	28 (18.1%)
Lumbago	23 (14.8%)
Anesthesia (n, %)	
Femoral nerve block	82 (52.9%)
Ankle block	73 (47.1%)

**Fig. 6:**
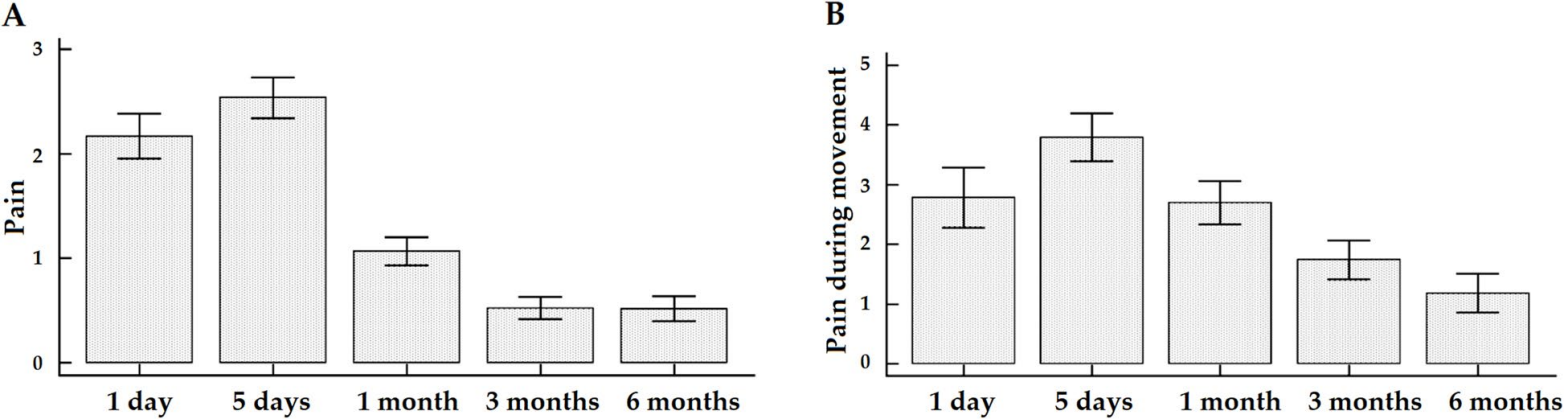
Graphs showing the patients’ postoperative pain levels, at rest (**A**) and during movement (**B**), at different time-points until 6-month follow-up

**Fig. 7:**
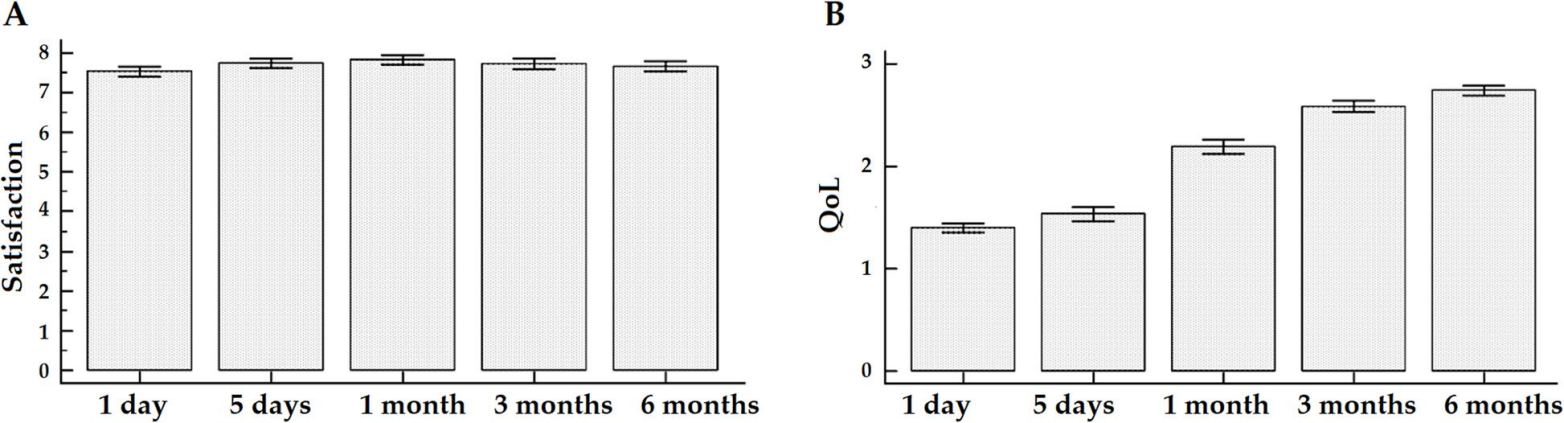
Graphs showing the patients’ postoperative satisfaction values (**A**), and quality of life values (**B**) at different time-points until 6-month follow-up

Pain at rest significantly decreased from 2.17 at the first post-operative day to 0.52 at 6 months (Fig. 6A, F = 44.43, *p* < 0.0001), as well as pain during movement from 2.79 to 1.18 (Fig. 6B, F = 36.26, *p* < 0.0001). For both measures, all time-points were significant at the post-hoc pairwise comparison analysis except for the comparison between the measurement at 1 and 5 days after the operation and between 3 and 6 months for pain at rest and between 1 post-operative day and the 1-month point as well as between 3 and 6 months for pain during movement. At 3 and 6 months, 11 (7.1%) and 13 (8.4%) patients suffered from CPS, respectively. Satisfaction remained stable at the different time-points (Fig. 7A, F = 1.53, *p* > 0.05), whereas quality of life significantly increased from 1.40 to 2.74 (Fig. 7B, F = 151.24, *p* < 0.0001). All time-points were significant at the post-hoc pairwise comparison analysis except for the comparison between the 1 and the 5 post-operative days as well as between 3 and 6 months after the operation (Table 2). At the different time-points, 1 (0.6%), 15 (9.7%), 93 (60.0%), 140 (90.3%), and 147 (94.8%) patients gradually returned to their daily activities and previous employment (*p* < 0.0001).

**Table 2:** Major outcomes at different post-operative time-points of the analyzed cohort

Variable	Mean	SD	95% CI	Statistical significance
**Pain at rest**				
1 day	2.17	0.22	1.74 to 2.60	Significantly different from 3, 4, 5
5 days	2.54	0.20	2.15 to 2.92	Significantly different from 3, 4, 5
1 month	1.06	0.14	0.80 to 1.33	Significantly different from 1, 2, 4, 5
3 months	0.52	0.11	0.31 to 0.73	Significantly different from 1, 2, 3
6 months	0.52	0.12	0.28 to 0.75	Significantly different from 1, 2, 3
**Pain during movement**				
1 day	2.79	0.25	2.28 to 3.29	Significantly different from 2, 4, 5
5 days	3.79	0.20	3.39 to 4.20	Significantly different from 1, 3, 4, 5
1 month	2.70	0.19	2.33 to 3.06	Significantly different from 2, 4, 5
3 months	1.74	0.16	1.42 to 2.07	Significantly different from 1, 2, 3
6 months	1.18	0.16	0.86 to 1.50	Significantly different from 1, 2, 3
**Satisfaction**				
1 day	7.53	0.13	7.28 to 7.78	Not significantly different from 2, 3, 4, 5
5 days	7.75	0.12	7.52 to 7.98	Not significantly different from 1, 3, 4, 5
1 month	7.83	0.12	7.59 to 8.07	Not significantly different from 1, 2, 4, 5
3 months	7.73	0.13	7.47 to 7.99	Not significantly different from 1, 2, 3, 5
6 months	7.66	0.13	7.40 to 7.93	Not significantly different from 1, 2, 3, 4
**Quality of life**				
1 day	1.40	0.05	1.31 to 1.49	Significantly different from 3, 4, 5
5 days	1.53	0.06	1.40 to 1.66	Significantly different from 3, 4, 5
1 month	2.19	0.07	2.05 to 2.33	Significantly different from 1, 2, 4, 5
3 months	2.58	0.06	2.47 to 2.70	Significantly different from 1, 2, 3
6 months	2.74	0.05	2.65 to 2.83	Significantly different from 1, 2, 3

No overall impact of type of anaesthesia (sciatic-femoral nerve block versus ankle block) on the outcomes could be detected (Table 3). Pain at rest on the fifth day was higher among those with the femoral nerve block with respect to those with the ankle block (*p* = 0.034). Perceived quality of life on the fifth day also differed between the two groups, being higher among those with ankle block (*p* = 0.041). However, when correcting for multiple comparisons, these small differences failed to achieve statistical significance. Further, other variables under study did not impact major outcomes apart from the ASA classification (*p* = 0.043) with higher movement with pain values reported in the ASA 3 group, and BMI (*p* = 0.005) and lumbago (*p* = 0.004), with lower satisfaction values (Table 4). Finally, no complications relative to both regional anaesthesia procedures were recorded, such as postoperative neuropathic symptoms, nerve injuries or systemic adverse events.

**Table 3:** Main characteristics of the recruited sample of 155 patients broken down according to the type of anesthesia

Parameters	Sciatic-Femoral nerve block (82 patients)	Ankle block (73 patients)	***P***-value
Age (years)	57.98 ± 12.71	60.16 ± 11.60	0.267
BMI (kg/m^2^)	27.13 ± 5.08	26.59 ± 4.61	0.493
Sex (n, %)			0.450
Male	69 (84.1%)	58 (79.5%)	
Female	13 (15.9%)	15 (20.5%)	
ASA classification (n, %)			0.841
1	26 (31.7%)	21 (28.8%)	
2	49 (59.8%)	44 (60.3%)	
3	7 (8.5%)	8 (11.0%)	
Risk factors (n, %)			
Preoperative Pain	42 (51.2%)	31 (42.5%)	0.277
Anxiety-depression	14 (17.1%)	10 (13.7%)	0.563
Inflammation	4 (4.9%)	3 (4.1%)	0.819
Obesity	17 (20.7%)	11 (15.1%)	0.362
Lumbago	11 (13.4%)	12 (16.4%)	0.598
**Pain at rest**			
1 day	2.50 ± 2.85	1.79 ± 2.48	0.104
5 days	2.93 ± 2.52	2.10 ± 2.29	**0.034**
1 month	1.07 ± 1.62	1.05 ± 1.76	0.946
3 months	0.54 ± 1.21	0.51 ± 1.45	0.890
6 months	0.52 ± 1.44	0.51 ± 1.50	0.941
**Pain during movement**			
1 day	2.87 ± 3.17	2.70 ± 3.19	0.744
5 days	3.76 ± 2.54	3.84 ± 2.57	0.847
1 month	2.43 ± 2.17	3.00 ± 2.44	0.124
3 months	1.84 ± 2.11	1.63 ± 1.97	0.521
6 months	1.46 ± 2.36	0.86 ± 1.58	0.068
**Satisfaction**			
1 day	7.45 ± 1.54	7.62 ± 1.64	0.519
5 days	7.57 ± 1.56	7.95 ± 1.33	0.114
1 month	7.79 ± 1.60	7.88 ± 1.44	0.733
3 months	7.76 ± 1.75	7.70 ± 1.54	0.829
6 months	7.73 ± 1.66	7.59 ± 1.63	0.591
**Quality of life**			
1 day	1.35 ± 0.57	1.45 ± 0.55	0.281
5 days	1.41 ± 0.79	1.67 ± 0.80	**0.041**
1 month	2.12 ± 0.87	2.27 ± 0.89	0.282
3 months	2.54 ± 0.74	2.63 ± 0.66	0.472
6 months	2.74 ± 0.58	2.74 ± 0.58	0.964

**Table 4:** Impact of variables under study on major outcomes of the analyzed cohort

Source	F	***P Value***	***η***_**p**_^**2**^
**Pain at rest**			
Intercept	0.66	0.418	0.005
Age	0.00	0.970	0.000
Sex	0.00	0.986	0.000
BMI	0.48	0.490	0.003
ASA	0.03	0.968	0.000
Anesthesia	1.93	0.167	0.013
Pain	2.16	0.144	0.015
Anxiety-depression	0.09	0.760	0.001
Inflammation	0.19	0.661	0.001
Lumbago	0.07	0.799	0.000
**Pain during movement**			
Intercept	19.26	0.000	0.119
Age	2.85	0.094	0.020
Sex	0.23	0.635	0.002
BMI	2.32	0.130	0.016
ASA	3.22	**0.043**	0.043
Anesthesia	0.03	0.858	0.000
Pain	0.69	0.409	0.005
Anxiety-depression	0.66	0.416	0.005
Inflammation	0.05	0.821	0.000
Lumbago	0.15	0.695	0.001
**Satisfaction**			
Intercept	49.87	0.000	0.259
Age	0.09	0.766	0.001
Sex	0.51	0.478	0.004
BMI	8.32	**0.005**	0.055
ASA	0.79	0.457	0.011
Anesthesia	0.55	0.460	0.004
Pain	0.37	0.544	0.003
Anxiety-depression	0.71	0.399	0.005
Inflammation	2.31	0.131	0.016
Lumbago	8.70	**0.004**	0.057
**Quality of life**			
Intercept	44.51	0.000	0.239
Age	0.68	0.412	0.005
Sex	1.48	0.226	0.010
BMI	0.15	0.702	0.001
ASA	1.05	0.352	0.015
Anesthesia	2.45	0.120	0.017
Pain	1.79	0.183	0.012
Anxiety-depression	0.06	0.809	0.000
Inflammation	0.25	0.620	0.002
Lumbago	2.61	0.109	0.018

At the multivariate logistic regression analysis, no statistically significant predictors of CPS could be detected at 3 (Table 5) and 6 months (Table 6).

**Table 5:** Multivariate logistic regression analysis shedding light on the determinants of the insurgence of CPS at 3 months

Variable	Coefficient	Standard error	Wald	*p-value*	Odds ratio	95%CI
Age	0.00	0.03	0.01	0.9406	1.00	0.94 to 1.07
BMI	−0.15	0.11	1.70	0.1921	0.87	0.70 to 1.08
Sex	−1.23	0.77	2.58	0.1080	0.29	0.06 to 1.31
ASA classification 2 (vs 1)	1.94	1.17	2.73	0.0985	6.94	0.70 to 69.10
Risk factors:						
Preoperative Pain	−1.06	0.76	1.95	0.1625	0.35	0.08 to 1.54
Anxiety-depression	0.33	0.91	0.13	0.7142	1.40	0.23 to 8.37
Obesity	1.15	1.22	0.90	0.3424	3.17	0.29 to 34.31
Lumbago	−0.47	1.20	0.16	0.6916	0.62	0.06 to 6.50
Anaesthesia	0.30	0.69	0.19	0.6629	1.35	0.35 to 5.24
Constant	0.73	3.13	0.05	0.8167

**Table 6:** Multivariate logistic regression analysis shedding light on the determinants of the insurgence of CPS at 6 months

Variable	Coefficient	Standard error	Wald	*p-value*	Odds ratio	95%CI
Age	0.03	0.03	0.70	0.4023	1.03	0.96 to 1.10
BMI	−0.04	0.09	0.19	0.6660	0.96	0.80 to 1.15
Sex	1.37	1.11	1.52	0.2170	3.93	0.45 to 34.54
ASA classification						
2 (vs 1)	1.01	0.88	1.32	0.2509	2.75	0.49 to 15.49
3 (vs 1)	0.62	1.37	0.21	0.6484	1.86	0.13 to 27.14
Risk factors:						
Preoperative Pain	−1.16	0.68	2.96	0.0853	0.31	0.08 to 1.18
Anxiety-depression	−0.38	0.87	0.19	0.6659	0.69	0.13 to 3.77
Obesity	−0.18	1.15	0.02	0.8762	0.84	0.09 to 7.91
Lumbago	−0.52	0.87	0.36	0.5492	0.59	0.11 to 3.26
Anaesthesia	−0.52	0.66	0.64	0.4254	0.59	0.16 to 2.14
Constant	−4.81	3.04	2.50	0.1137		

## Discussion

Operative procedures of the forefoot usually cause moderate to severe acute pain that can occasionally progress into CPS [35]. For these reasons, inadequate postoperative pain management in patients having undergone HV percutaneous correction in outpatient surgery can have several adverse outcomes, such as length of hospital stay, precipitated withdrawal and overall increase in health care costs.

While several studies have focused on the development of CPS after knee and hip surgeries [36–38], the literature still lacks studies concerning postoperative pain and CPS in foot and forefoot surgery and its prevalence after HV percutaneous correction. Studies on HV report mostly functional scores to describe clinical outcomes obtained after surgery.

Hence, the aims of this prospective study were to investigate the postoperative pain and CPS in a cohort of patients having undergone the same percutaneous operative procedure for HV correction, performed under ultrasound-guided *sciatic-femoral block* or *ankle-block*. Specifically, the impact of these types of anaesthetic blocks and risk factors on the development of postoperative pain, patient satisfaction and quality of life were evaluated.

The most important findings of the present study, observed from the first day to 6-month follow-up after surgery were as follows: a significant decrease of pain at rest and during movement; a stable level of patient satisfaction; a significant increase of patient quality of life and return to daily activities and work. Importantly, no significant impact of type of anaesthesia could be detected. ASA 3 was associated to higher pain during movement, while BMI and lumbago to lower patient satisfaction. Among risk factors, only a higher ASA was associated to higher pain during movement, while higher BMI and lumbago to lower satisfaction.

Both types of pain improved over time (from 1 day to 6 months after surgery) as well as the quality of life, in accordance with the literature [39, 40]. Patient satisfaction did not change over time, and the high satisfaction rate observed was in agreement with data reported for the use of regional anaesthesia [41, 42]. The percentage of patients who developed CPS (NRS ≥ 4) was 7.1 and 8.4% at 3 and 6 months after surgery. These findings are acceptable considering that the surgical sites of feet are constantly solicited during daily activities. It should be underlined that for this report, the use of the NRS scale to evaluate pain was chosen as it is easier to administer and manage both verbally and in writing compared to the VAS scale [43].

The impact of risk factors and the type of anaesthesia (*femoral-sciatic* versus *ankle block*) on pain, pain during movement, satisfaction and quality of life was also analysed, finding that the block type does not have any influence on clinical outcomes. Many studies have compared *ankle blocks* to more proximal blocks [42, 44, 45] or compared the analgesic efficacy of an *ankle block* in addition to general anaesthesia or spinal anaesthesia [46, 47]. Only one study, by Tharwa et al., compared the efficacy and safety of *ankle block* versus *sciatic-saphenous nerve block* in 42 patients with HV having undergone surgery [48]. No difference was found comparing the efficacy and safety between the two blocks, but they observed a statistically significant difference in the VAS pain score in the 12-h postoperative period, with ankle block showing higher pain levels requiring more postoperative pain killers [48]. The authors concluded that both blocks provided good intraoperative anaesthesia and satisfactory postoperative pain controls. However, they did not show a follow-up of these patients, making comparison with our data difficult.

In general, the relationship between ASA classes and postoperative pain has been poorly studied, and no studies about the impact of percutaneous HV procedures on pain after regional blocks have been published to date. On the contrary, the ASA scale used for this analysis was relevant, not only to objectively define the physical status of each enrolled patient before surgery, reducing the potential inter-observer variability classification of our cohort, but also to better correlate its preoperative health level with postoperative pain. In particular, we found that higher ASA had a major impact on pain during movement. A likely explanation of this finding is that patients with higher ASA are more prone to have other diseases and co-existent pain [49] despite the exclusion criteria proposed for this study. The ASA 1 and 2 patients represented 90% of our cohort, reflecting a slight difference between groups in terms of major comorbidity (ASA 3:10%).

We also identified an association between a lower satisfaction with BMI and lumbago. HV has been reported to be inversely associated with obesity [50], and only Wirth et al. reported no evidence of an association of improvable patient satisfaction with BMI in patients treated surgically for HV, but no data about the anaesthesia used were reported [51]. In line with our results, Hegewald and colleagues demonstrated that patient age and BMI contribute to the differences in overall block outcome with more successful blocks observed in patients with a lower BMI [52]. Chen et al. [53] compared the clinical outcomes of obese patients with normal weight patients treated surgically for HV, and no differences were found.

The association between satisfaction and lumbago is not surprising, as it has been reported that both foot and ankle deviation could be a potential cause of low-back pain due to the disruption of the kinetic chain from the foot to the back [54].

In our study, the presence of preoperative pain was not related to development of postoperative pain, while it has been reported that the presence of preoperative pain is correlated to the development of chronic neuropathic pain [2]. Generally, inadequate treatment of acute pain represents a critical risk factor for the development of chronic pain, and persistent pain is suggested to influence procedure-related functional outcomes [55]. Chen et al. found that a higher preoperative VAS pain increased the risk of having some degree of residual pain at 6 months after surgery in a cohort of 317 patients who underwent HV surgery for pain and deformity [19]. However, it should be specified that in our study, although all HV treated were symptomatic, we recorded the presence of preoperative pain in less than 50% of our patients without using VAS scores, which could explain this low percentage with respect to those reported in the literature [2, 19, 55]. Probably, the preoperative recording of VAS scores among our patients would not have reached those reported previously, our subjects having mild-to-moderate HV deformity and often complaining about pain only during some daily activities.

Further, depression, anxiety and pre-existing inflammatory states were not associated with pain, quality of life and patient satisfaction. This may be related to the low number of patients affected by these risk factors in our cohort. In 2016, some factors of socioeconomic status (unemployment, poverty and no health insurance coverage) were reported to be common elements in promoting a high-impact chronic pain prevalence on the USA population [56]. For this study however, different risk factors were selected because national health care and economic unemployment support are guaranteed, so socioeconomic status is not a problem. Consistent with the literature [46], we did not observe anaesthesia complications, supporting the use of regional anaesthesia, which has several advantages including improved patient satisfaction, faster mobilisation, reduced length of hospital stay and reduced used of opioids [41, 57].

An inadequate perioperative anaesthesia and unsatisfactory postoperative pain control protocol may lead to the development of CPS, inducing the use of opioids in postoperative therapy, and sometimes the consequent development of an opioid use disorder (OUD), which can compromise pain management also in the case of future operations. Parrish JM and colleagues [58] demonstrated that patients with a history of OUD undergoing hallux valgus correction had higher odds of 90-day readmission rates and 30-day Emergency Room visits. Further, patients with a history of OUD demonstrated a higher 90-day total global episode-of-care cost compared with those without OUD. Our patients, closely following the postoperative protocol did not need to resort to opioid use, which is reported to be greater in chronic pain patients due to tolerance, dependence and opioid-induced hyperalgesia [59]. For these reasons, orthopaedic surgeons should be aware that long-term postoperative opioid use must be avoided [59], as its inadvertent overprescription may place patients and their communities at risk of abuse or OUD [58, 60].

### Strengths and weaknesses

The strengths of our study include: (1) the standardization of anaesthesiology, operative procedures and postoperative pain therapy including aftercare, according to our institutional protocol for the same percutaneous operation – the first performed by the same team of anaesthesiologists, the second by the senior surgeon, the third in use at our institution since 2009; these aspects avoid confounding bias and allow adequate methodology for comparative reasons; (2) the prospective data collection of the case series with the same fixed follow-ups using validated questionnaires; (3) an adequate number of patients in both groups – none was lost at different follow-up points until the last one, and the appropriate power calculations were conducted for the primary outcome measures; (4) the analysis of the clinical outcomes, carried out separately by independent investigators; the person who performed clinical assessment was blinded to the type of procedure used; (5) the multivariable statistical analysis performed by an independent statistician.

We are also aware of the study’s weaknesses. (1) It was a single centre case series study with the same team of anaesthesiologists and a single surgeon for all operations; these aspects could have affected the generalisability of the operative procedure. (2) There was a lack of randomisation with potential selection biases, although the patients were operated during the 2-year study period alternating weekly one or the other regional anaesthesia block according to our study protocol and without any regional block preference by the anaesthetists. (3) We lacked a control group, which prevented us from comparing results. (4) The mere inclusion of cases of unilateral HV treated percutaneously prevented us from reporting outcomes of cases operated bilaterally or by more traditional open techniques. (5) Multivariate analysis was performed, but no determinants were found, probably because of the small number of CPS subjects. It would require a larger number of individuals. In our study, we found that only 11 (7.1%) and 13 (8.4%) patients suffered from CPS at 3 and 6 months, respectively.

Further larger studies aimed at identifying the determinants underlying the occurrence of CPS are needed.

## Conclusions

Our data show that postoperative pain at rest and during movement improved from the first day to 6-month follow-up after percutaneous HV correction, independently of the regional blocks performed and without postoperative complications of anaesthesia.

Supported by a tested institutional aftercare therapy protocol, both sciatic-femoral and ankle blocks were safe and effective in reducing postoperative pain with low incidence of CPS at last follow-up. The ultrasound-guided peripheral blocks were well suited to forefoot outpatient surgery settings, showed high patient acceptance rates and allowed improvement of quality of life and return to daily activities and work.

Finally, in relation to the different risk factors analysed, only a higher ASA was associated with pain during movement, while higher BMI values and the presence of lumbago were associated with lower satisfaction values.

## Data Availability

The dataset supporting the conclusions of this article is available at our institution contacting the corresponding author.

## Abbreviations

ASA: American Society of Anesthesiologists
BMI: Body mass index
CPS: Chronic pain syndrome
HV: Hallux valgus
MIS: Minimally invasive surgery
NRS: Numerical rating scale
VAS: Visual analogue scale

## References

[1] Bruce J, Quinlan J (2011). Chronic post surgical pain. Rev Pain.

[2] Kehlet H, Jensen TS, Woolf CJ (2006). Persistent postsurgical pain: risk factors and prevention. Lancet.

[3] Crombie IK, Davies HT, Macrae WA (1998). Cut and thrust: antecedent surgery and trauma among patients attending a chronic pain clinic. Pain.

[4] Macrae WA (2001). Chronic pain after surgery. Br J Anaesth.

[5] Schug SA, Lavand'homme P, Barke A, Korwisi B, Rief W, Treede RD (2019). The IASP classification of chronic pain for ICD-11: chronic postsurgical or posttraumatic pain. Pain.

[6] Gerbershagen HJ, Rothaug J, Kalkman CJ, Meissner W (2011). Determination of moderate-to-severe postoperative pain on the numeric rating scale: a cut-off point analysis applying four different methods. Br J Anaesth.

[7] Thapa P, Euasobhon P (2018). Chronic postsurgical pain: current evidence for prevention and management. Korean J Pain.

[8] Rüsch D, Eberhart LH, Wallenborn J, Kranke P (2010). Nausea and vomiting after surgery under general anesthesia: an evidence-based review concerning risk assessment, prevention, and treatment. Deutsches Arzteblatt Int.

[9] Benzon HT, Asher YG, Hartrick CT (2016). Back pain and neuraxial anesthesia. Anesth Analg.

[10] Jabbari A, Alijanpour E, Mir M, Bani Hashem N, Rabiea SM, Rupani MA (2013). Post spinal puncture headache, an old problem and new concepts: review of articles about predisposing factors. Caspian J Internal Med.

[11] Niazi AA, Taha MA (2015). Postoperative urinary retention after general and spinal anesthesia in orthopedic surgical patients. Egypt J Anaesth.

[12] Ogilvy AJ, Smith G (1995). The gastrointestinal tract after anaesthesia. Eur J Anaesthesiol Suppl.

[13] Vadivelu N, Kai AM, Maslin B, Kodumudi V, Antony S, Blume P (2015). Role of regional anesthesia in foot and ankle surgery. Foot Ankle Specialist.

[14] Pearce CJ, Hamilton PD (2010). Current concepts review: regional anesthesia for foot and ankle surgery. Foot Ankle Int.

[15] Clough TM, Sandher D, Bale RS, Laurence AS (2003). The use of a local anesthetic foot block in patients undergoing outpatient bony forefoot surgery: a prospective randomized controlled trial. J Foot Ankle Surg.

[16] Kang C, Hwang DS, Kim YM, Kim PS, Jun YS (2010). Ultrasound-guided femorosciatic nerve block by Orthopaedist for ankle fracture operation. JKFAS.

[17] Stein BE, Srikumaran U, Tan EW, Freehill MT, Wilckens JH (2012). Lower-extremity peripheral nerve blocks in the perioperative pain management of orthopaedic patients: AAOS exhibit selection. J Bone Joint Surg Am.

[18] Schipper ON, Hunt KJ, Anderson RB, Davis WH, Jones CP, Cohen BE (2017). Ankle block vs single-shot popliteal fossa block as primary anesthesia for forefoot operative procedures: prospective, randomized comparison. Foot Ankle Int.

[19] Chen JY, Ang BFH, Jiang L, Yeo NEM, Koo K, Singh RI (2016). Pain resolution after hallux valgus surgery. Foot Ankle Int.

[20] Chou LB, Wagner D, Witten DM, Martinez-Diaz GJ, Brook NS, Toussaint M, Carroll IR (2008). Postoperative pain following foot and ankle surgery: a prospective study. Foot Ankle Int.

[21] Ying J, Xu Y, István B, Ren F. Adjusted indirect and mixed comparisons of conservative treatments for hallux valgus: a systematic review and network meta-analysis. Int J Environ Res Public Health. 2021;18(7):3841. 10.3390/ijerph18073841 PMC8038851 33917568

[22] Biz C, Fosser M, Dalmau-Pastor M, Corradin M, Rodà MG, Aldegheri R, Ruggieri P (2016). Functional and radiographic outcomes of hallux valgus correction by mini-invasive surgery with Reverdin-Isham and Akin percutaneous osteotomies: a longitudinal prospective study with a 48-month follow-up. J Orthop Surg Res.

[23] Coughlin MJ, Mann RA, Saltzman CL (2007). Surgery of the foot and ankle.

[24] Dhukaram V, Kumar CS (2004). Nerve blocks in foot and ankle surgery. Foot Ankle Surg.

[25] Schurmax DJ (1976). Ankle-block anesthesia for foot surgery. J Am Soc Anesthesiol.

[26] de Prado M, Ripoll P-L, Golanó P, Maffulli N, Easley M (2011). Minimally invasive Management of Hallux Rigidus. Minimally invasive surgery of the foot and ankle.

[27] Biz C, Corradin M, Petretta I, Aldegheri R (2015). Endolog technique for correction of hallux valgus: a prospective study of 30 patients with 4-year follow-up. J Orthop Surg Res.

[28] Biz C, Crimì A, Fantoni I, Tagliapietra J, Ruggieri P. Functional and radiographic outcomes of minimally invasive intramedullary nail device (MIIND) for moderate to severe hallux valgus. Foot Ankle Int. 2020:42(4):409–24. 10.1177/1071100720969676 33319594

[29] Gicquel T, Fraisse B, Marleix S, Chapuis M, Violas P (2013). Percutaneous hallux valgus surgery in children: short-term outcomes of 33 cases. Orthop Traumatol Surg Res.

[30] Pavan R, Jain S, Shraddha KA (2012). Properties and therapeutic application of bromelain: a review. Biotechnol Res Int.

[31] Mayhew D, Mendonca V, Murthy BVS (2019). A review of ASA physical status – historical perspectives and modern developments. Anaesthesia.

[32] Hackett NJ, De Oliveira GS, Jain UK, Kim JY (2015). ASA class is a reliable independent predictor of medical complications and mortality following surgery. Int J Surg.

[33] Kronzer VL, Jerry MR, Avidan MS (2016). Assessing change in patient-reported quality of life after elective surgery: protocol for an observational comparison study. F1000Research.

[34] Cohen J (1988). Statistical power analysis for the behavioural sciences.

[35] Needoff M, Radford P, Costigan P (1995). Local anesthesia for postoperative pain relief after foot surgery: a prospective clinical trial. Foot Ankle Int.

[36] Vergne-Salle P (2016). Management of neuropathic pain after knee surgery. Joint Bone Spine.

[37] Puolakka PA, Rorarius MG, Roviola M, Puolakka TJ, Nordhausen K, Lindgren L (2010). Persistent pain following knee arthroplasty. Eur J Anaesthesiol.

[38] Wylde V, Hewlett S, Learmonth ID, Dieppe P (2011). Persistent pain after joint replacement: prevalence, sensory qualities, and postoperative determinants. Pain.

[39] Saro C, Jensen I, Lindgren U, Felländer-Tsai L (2007). Quality-of-life outcome after hallux valgus surgery. Qual Life Res.

[40] Zhu M, Chen JY, Yeo NEM, Koo K, Rikhraj IS (2020). Health-related quality-of-life improvement after hallux valgus corrective surgery. Foot Ankle Surg.

[41] Roberts VI, Aujla RS, Vinay S, Fombon FN, Singh H, Bhatia M (2020). Is regional ankle block needed in conjunction with general anaesthesia for first ray surgery? A randomised controlled trial of ultrasound guided ankle block versus “blind” local infiltration. Foot Ankle Surg.

[42] Migues A, Slullitel G, Vescovo A, Droblas F, Carrasco M, Turenne HP (2005). Peripheral foot blockade versus popliteal fossa nerve block: a prospective randomized trial in 51 patients. J Foot Ankle Surg.

[43] Hawker GA, Mian S, Kendzerska T, French M (2011). Measures of adult pain: Visual Analog Scale for Pain (VAS Pain), Numeric Rating Scale for Pain (NRS Pain), McGill Pain Questionnaire (MPQ), Short-Form Mcgill Pain Questionnaire (SF-MPQ), Chronic Pain Grade Scale (CPGS), Short Form-36 Bodily Pain Scale (SF-36 BPS), and Measure of Intermittent and Constant Osteoarthritis Pain (ICOAP). Arthritis Care Res.

[44] McLeod DH, Wong DH, Vaghadia H, Claridge RJ, Merrick PM (1995). Lateral popliteal sciatic nerve block compared with ankle block for analgesia following foot surgery. Can J Anaesth.

[45] Samuel R, Sloan A, Patel K, Aglan M, Zubairy A (2008). The efficacy of combined popliteal and ankle blocks in forefoot surgery. JBJS.

[46] Stéfani KC, Ferreira GF, Pereira Filho MV (2017). Postoperative analgesia using peripheral anesthetic block of the foot and ankle. Foot Ankle Int.

[47] Kir MC, Kir G (2018). Ankle nerve block adjuvant to general anesthesia reduces postsurgical pain and improves functional outcomes in hallux valgus surgery. Med Princ Pract.

[48] Tharwat A, El Shazly O (2014). Efficacy and safety of ankle block versus sciatic-saphenous nerve block for hallux valgus surgery. Ain-Shams J Anaesthesiol.

[49] Kinjo S, Sands LP, Lim E, Paul S, Leung JM (2012). Prediction of postoperative pain using path analysis in older patients. J Anesth.

[50] Dufour AB, Casey VA, Golightly YM, Hannan MT (2014). Characteristics associated with hallux valgus in a population-based foot study of older adults. Arthritis Care Res (Hoboken).

[51] Wirth SH, Renner N, Niehaus R, Farei-Campagna J, Deggeller M, Scheurer F, Palmer K, Jentzsch T (2019). The influence of obesity and gender on outcome after reversed L-shaped osteotomy for hallux valgus. BMC Musculoskelet Disord.

[52] Hegewald K, McCann K, Elizaga A, Hutchinson BL (2014). Popliteal blocks for foot and ankle surgery: success rate and contributing factors. J Foot Ankle Surg.

[53] Chen JY, Lee MJ, Rikhraj K, Parmar S, Chong HC, Yew AK, Koo KO, Singh RI (2015). Effect of obesity on outcome of hallux valgus surgery. Foot Ankle Int.

[54] O'Leary CB, Cahill CR, Robinson AW, Barnes MJ, Hong J (2013). A systematic review: the effects of podiatrical deviations on nonspecific chronic low back pain. J Back Musculoskel Rehabil.

[55] Sinatra R (2010). Causes and consequences of inadequate Management of Acute Pain. Pain Med.

[56] Dahlhamer J, Lucas J, Zelaya C, Nahin R, Mackey S, DeBar L, Kerns R, Von Korff M, Porter L, Helmick C (2018). Prevalence of chronic pain and high-impact chronic pain among adults - United States, 2016. MMWR Morb Mortal Wkly Rep.

[57] Joshi G, Gandhi K, Shah N, Gadsden J, Corman SL (2016). Peripheral nerve blocks in the management of postoperative pain: challenges and opportunities. J Clin Anesth.

[58] Parrish JM, Vakharia RM, Benson DC, Hoyt AK, Jenkins NW, Kaplan JRM, et al. Patients with opioid use disorder have increased readmission rates, emergency room visits, and costs following a hallux Valgus procedure. Foot Ankle Specialist. 2020:1938640020950105. Online ahead of print. 10.1177/1938640020950105 32857596

[59] Rogero R, Fuchs D, Nicholson K, Shakked RJ, Winters BS, Pedowitz DI, Raikin SM, Daniel JN (2019). Postoperative opioid consumption in opioid-naïve patients undergoing hallux valgus correction. Foot Ankle Int.

[60] Bhashyam AR, Keyser C, Miller CP, Jacobs J, Bluman E, Smith JT, Chiodo C (2019). Prospective evaluation of opioid use after adoption of a prescribing guideline for outpatient foot and ankle surgery. Foot Ankle Int.

